# Supplementary figures and images for: System-wide biochemical analysis reveals ozonide antimalarials initially act by disrupting Plasmodium falciparum haemoglobin digestion
Source: PLoS Pathog. 2020 Jun 26;16(6):e1008485. doi: 10.1371/journal.ppat.1008485 (PMC7347234; doi:10.1371/journal.ppat.1008485)

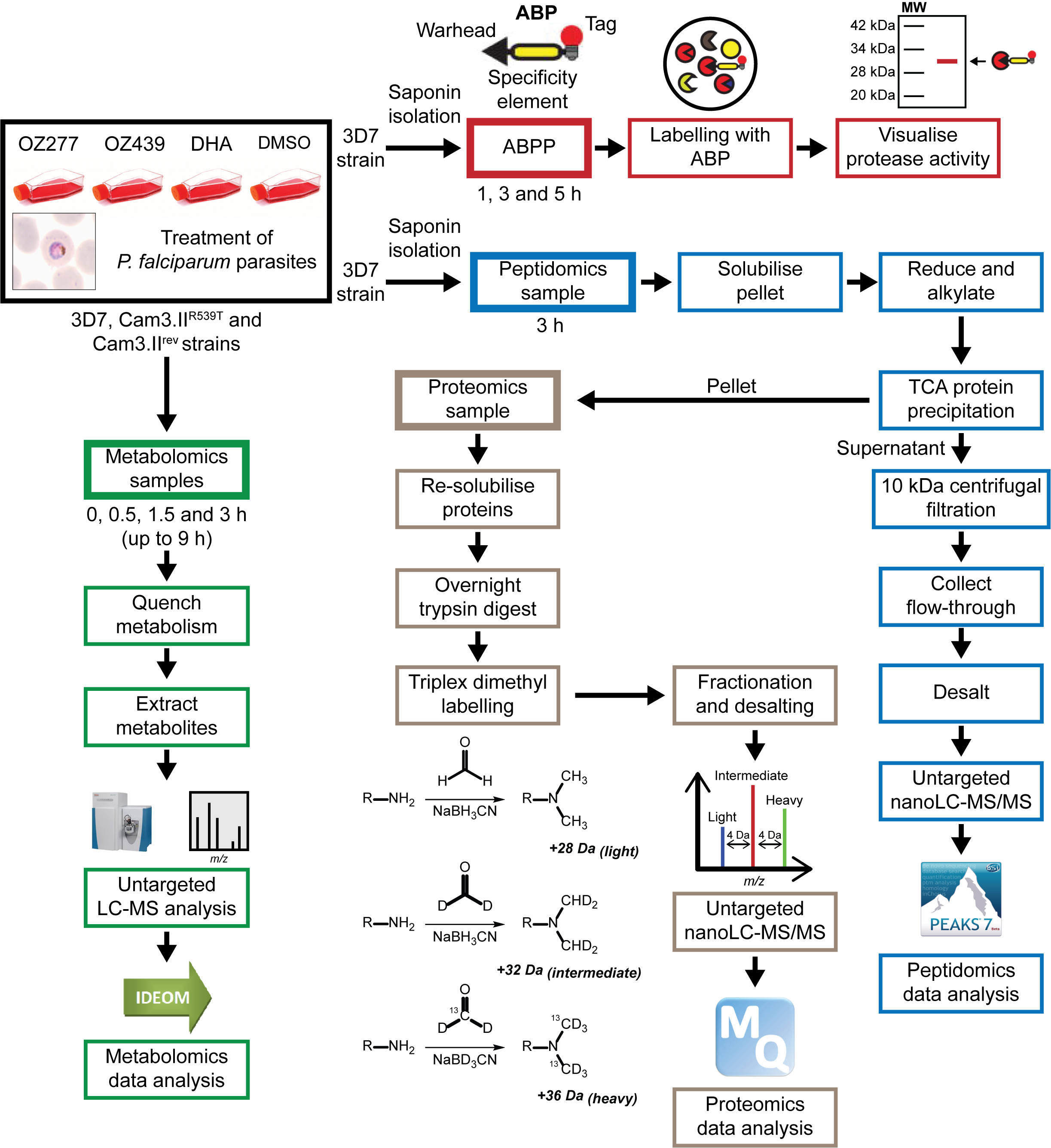

Supplement: S1 Fig — Metabolomics (green boxes) samples were extracted from infected RBCs at regular intervals (up to 9 h after drug addition) and the extracted samples were analysed by LC-MS and processed using IDEOM. For peptidomics (blue boxes), endogenous peptides were extracted from saponin-lysed parasites, samples were subjected to 10 kDa cut-off centrifugal filtration and then an equal amount of peptides were used for analysis by nanoLC-MS/MS before data was processed using PEAKS software. Protein pellets following trichloracetic acid (TCA) precipitation were collected for proteomic sample preparation (brown boxes). Equal amount of proteins were digested using trypsin, labelled using dimethyl labelling to allow differential quantitative proteomic analysis and examined using nanoLC-MS/MS followed by data processing using MaxQuant. ABPP assays (red boxes) were performed by selectively labelling active cysteine proteases in the parasite lysate using various activity-based probes (ABPs), then visualising protease activity in a gel-based format. (TIF) [file ppat.1008485.s001.tif]

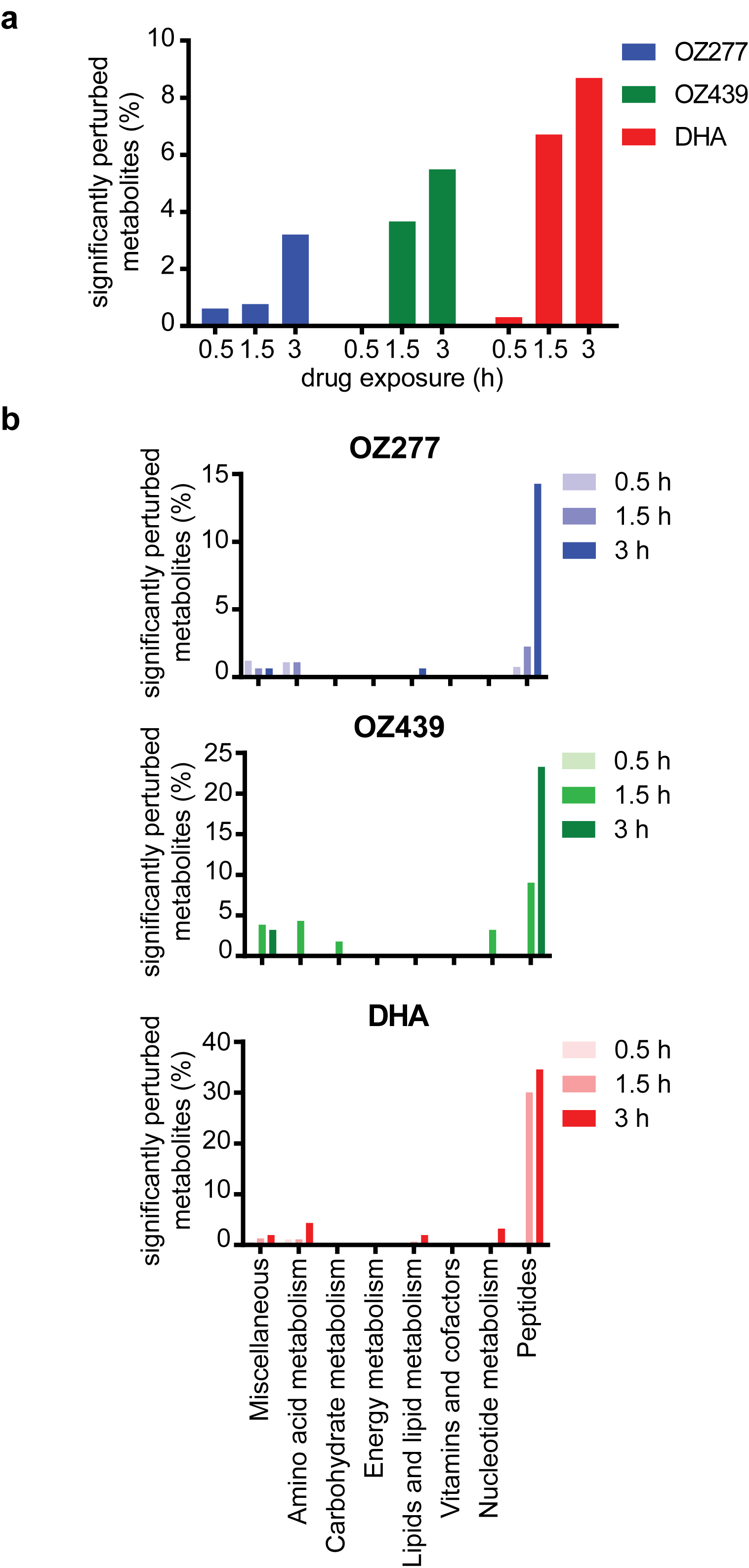

Supplement: S2 Fig — a, Percentage of significantly perturbed metabolites (Welch’s t test; P-value < 0.05 and fold change ≥ 1.5) following exposure to increasing durations of OZ277 (blue), OZ439 (green) and DHA (red) (0.5, 1.5 and 3 h). b, Pathway enrichment analysis showing the percentage of significantly perturbed metabolites (Welch’s t test; P-value < 0.05 and fold change > 1.5) as a function of metabolite class for short (0.5, 1.5 and 3 h) exposure with OZ277, OZ439 (both 300 nM) and DHA (100 nM). (TIF) [file ppat.1008485.s002.tif]

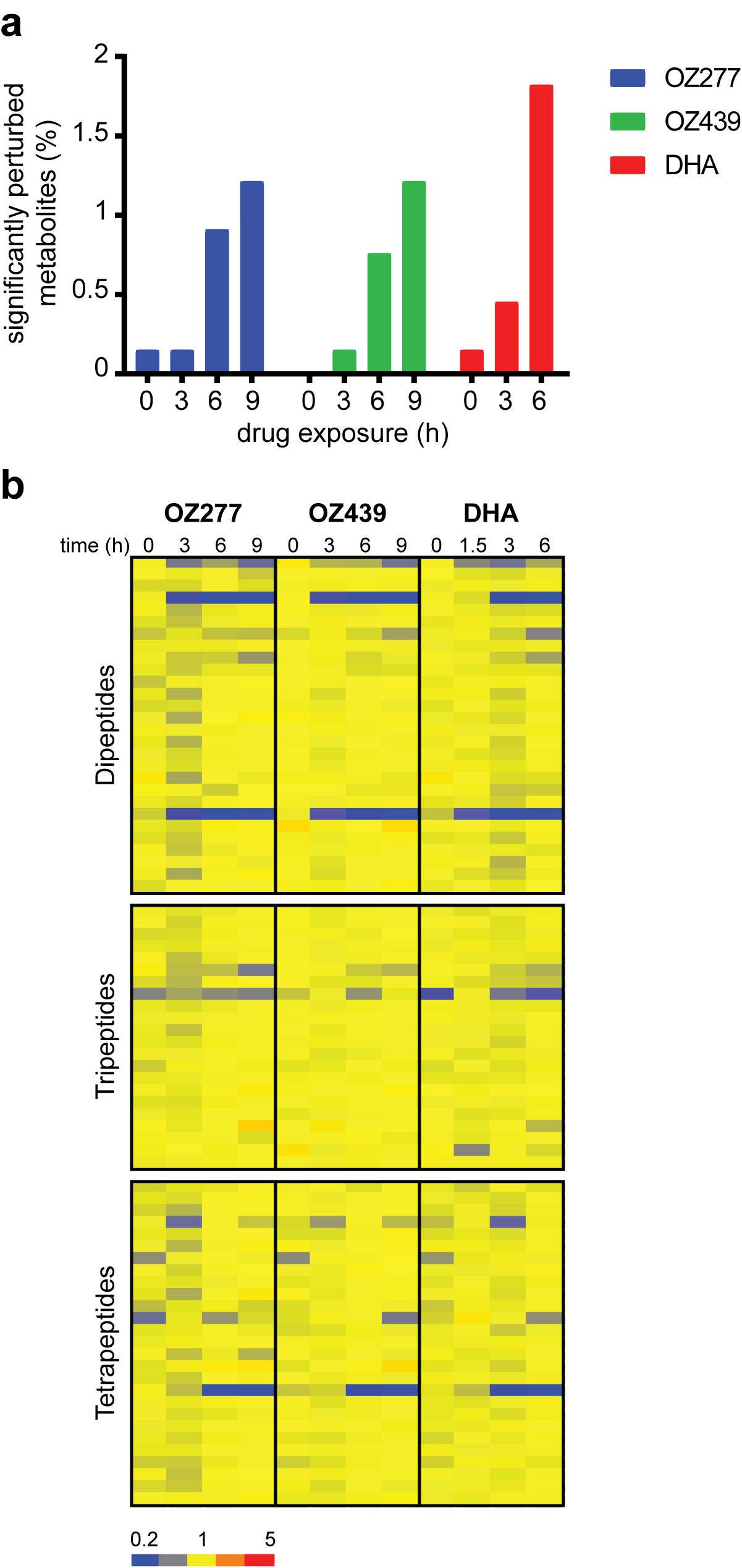

Supplement: S3 Fig — a, Percentage of significantly perturbed metabolites (Welch’s t test; P-value < 0.05 and fold-change > 1.5) at each time point (0, 3, 6 and 9 h) following OZ277 (blue), OZ439 (green) and DHA (red) treatment of ring-stage infected cultures. b, Heatmap showing the average fold change for all identified peptides at each time point after OZ277, OZ439 and DHA treatment in ring-stage parasites. For each time point, values represent the average of four independent biological replicates, expressed relative to the average untreated control value for that respective time point. (TIF) [file ppat.1008485.s003.tif]

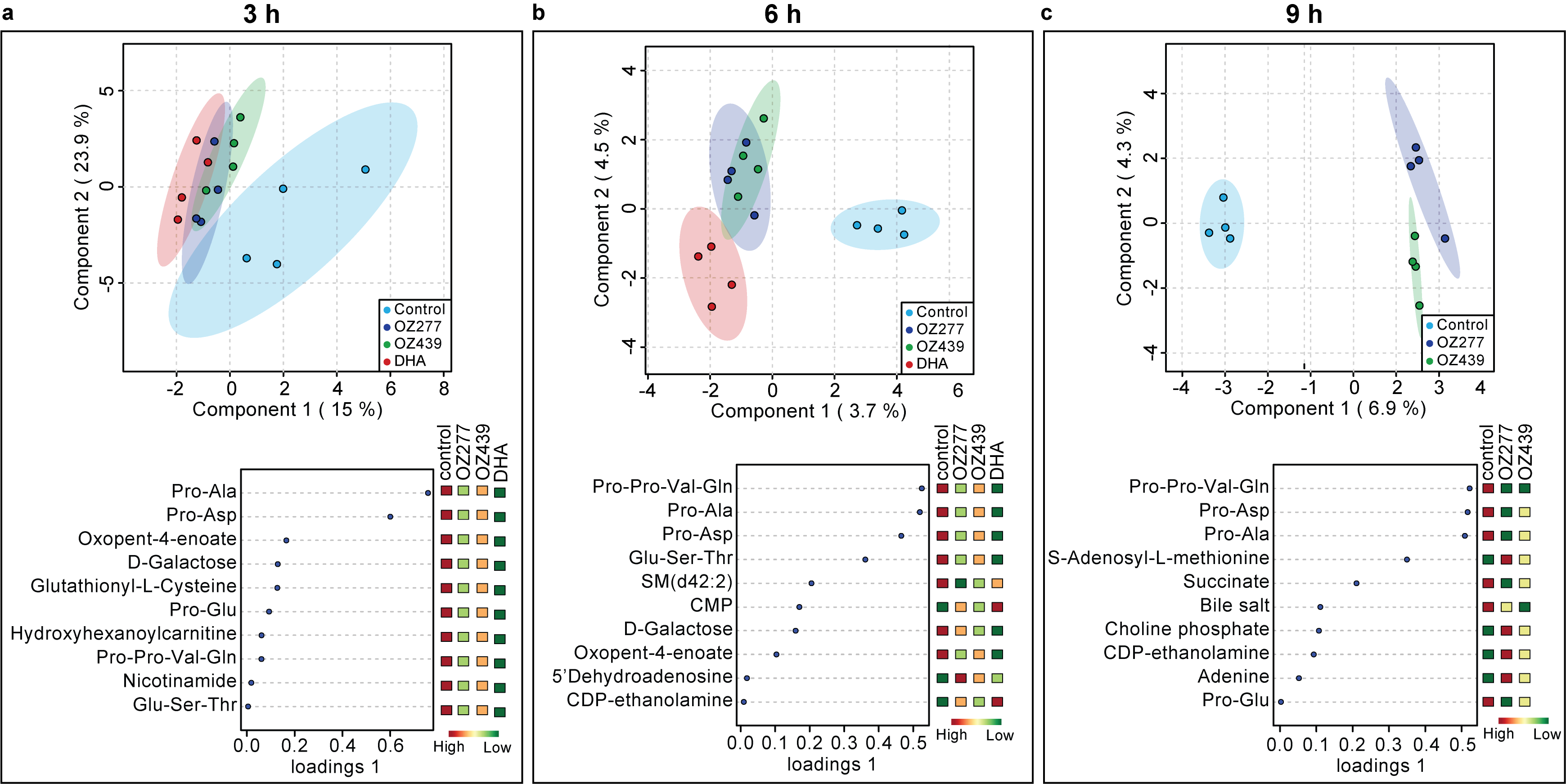

Supplement: S4 Fig — a-c, Sparse partial least squares–discriminant analysis (sPLS-DA) of the metabolomics data for ring-stage parasite infected RBCs treated with peroxide antimalarials for 3 h (a), 6 h (b), and 9 h (c). (TIF) [file ppat.1008485.s004.tif]

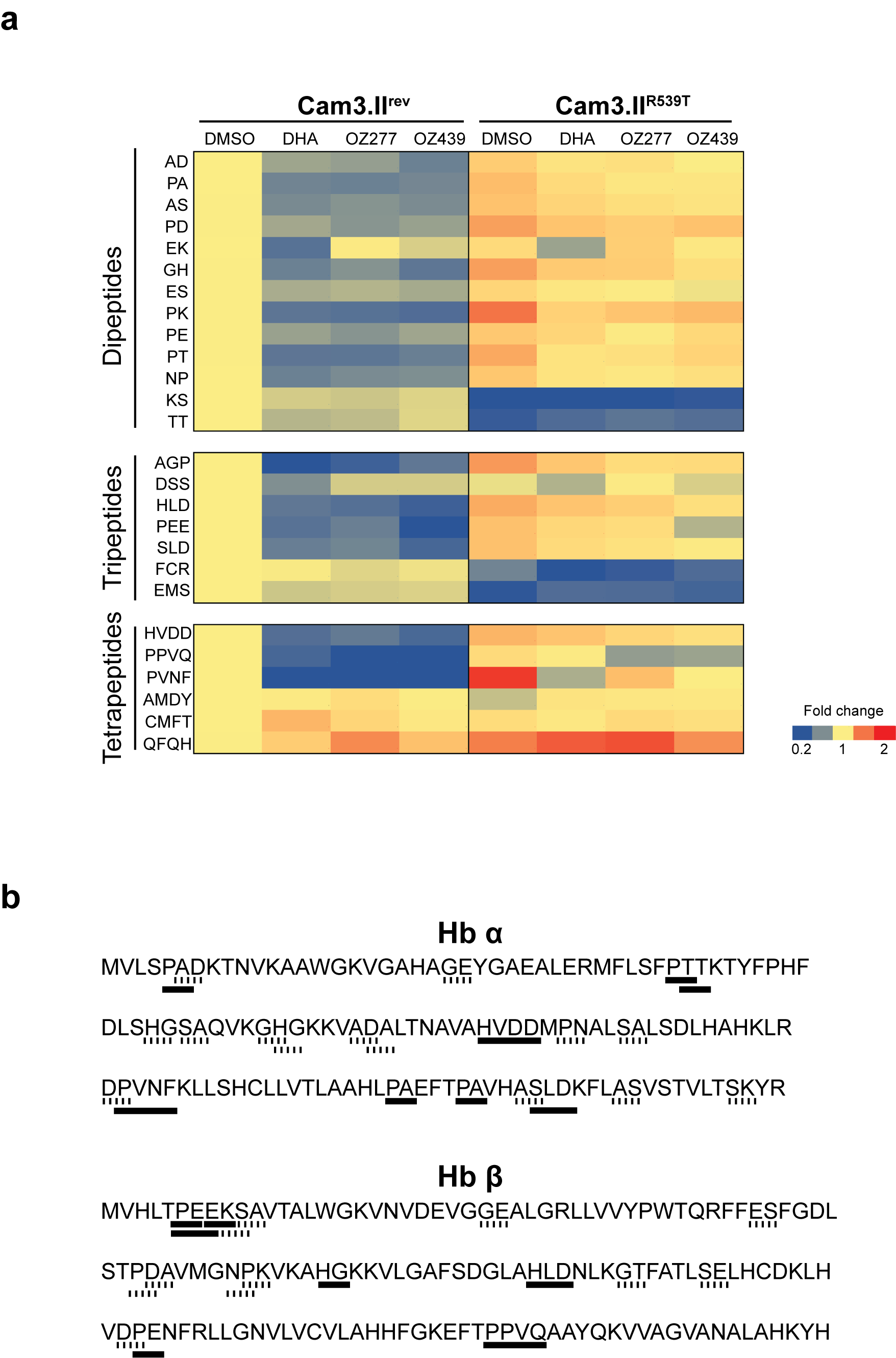

Supplement: S5 Fig — a, Heatmap of differentially abundant peptides (altered ≥ 1.5-fold relative to the untreated control) in artemisinin-resistant Cam3.IIR539T and artemisinin-sensitive Cam3.IIrev parasite lines following peroxide treatment. Data shown are the average for three or five biological replicates expressed relative to the average for the untreated control from the sensitive parasite line (DMSO, Cam3.IIrev).b, Sequence coverage for putative haemoglobin (Hb)-derived peptides that were altered in abundance following peroxide antimalarial treatment relative to the untreated control. All peptides, except TT and SK (or its isomers) were reduced in abundance in treated parasites compared to control levels. The solid black bars represent peptide sequences that were confirmed by MS/MS analysis and the dashed bars represent putative peptide sequences. For putative peptide sequences, all potential isomers have been mapped. (TIF) [file ppat.1008485.s005.tif]

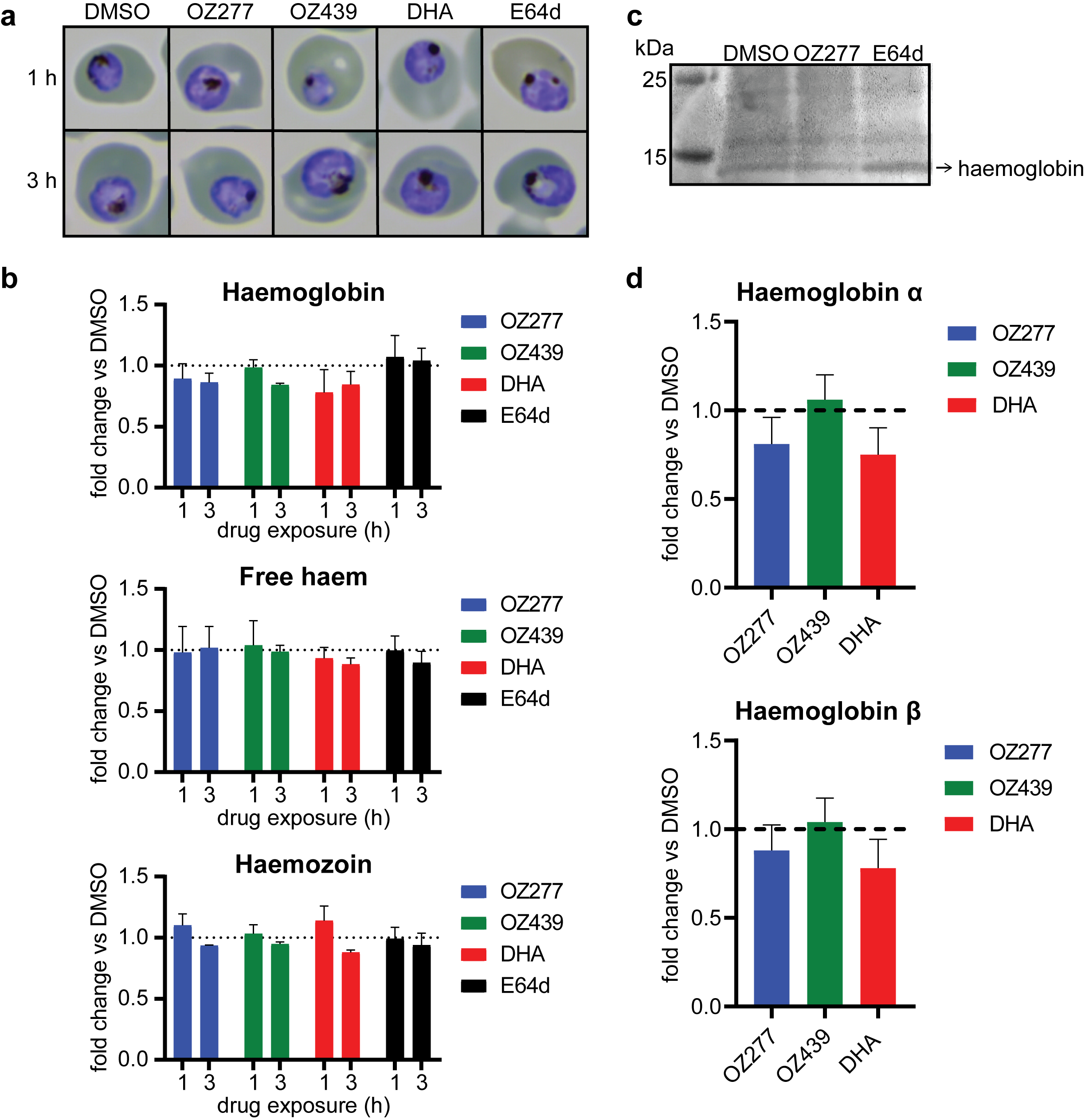

Supplement: S6 Fig — a, A panel of representative giemsa stained parasites treated with 300 nM of OZ277 or OZ439, 100 nM of DHA, a DMSO negative control or a 27 μM E64d positive control for a swollen digestive vacuole after 1 h and 3 h. b, Bar charts representing the relative levels of haemoglobin, free heme and hemozoin in trophozoite-stage parasites following either a 1 or 3 h incubation with 300 nM of OZ277 and OZ439, 100 nM of DHA or 27 μM of E64d expressed as the fold change when compared to a DMSO control. Bars represent the mean of >4 paired replicates from at least two independent experiments with the error bars expressed as SD. c, SDS-PAGE gel image of haemoglobin monomer band following treatment with OZ277 (300 nM for 3 h), E64d (27 μM for 3 h) and DMSO control. d, Haemoglobin α and β abundance measured using quantitative proteomics for treated parasites (100 nM DHA, 300 nM OZ277 and OZ439 for 3 h), values are the average fold change (± SD) relative to the untreated control of at least three biological replicates. (TIF) [file ppat.1008485.s006.tif]

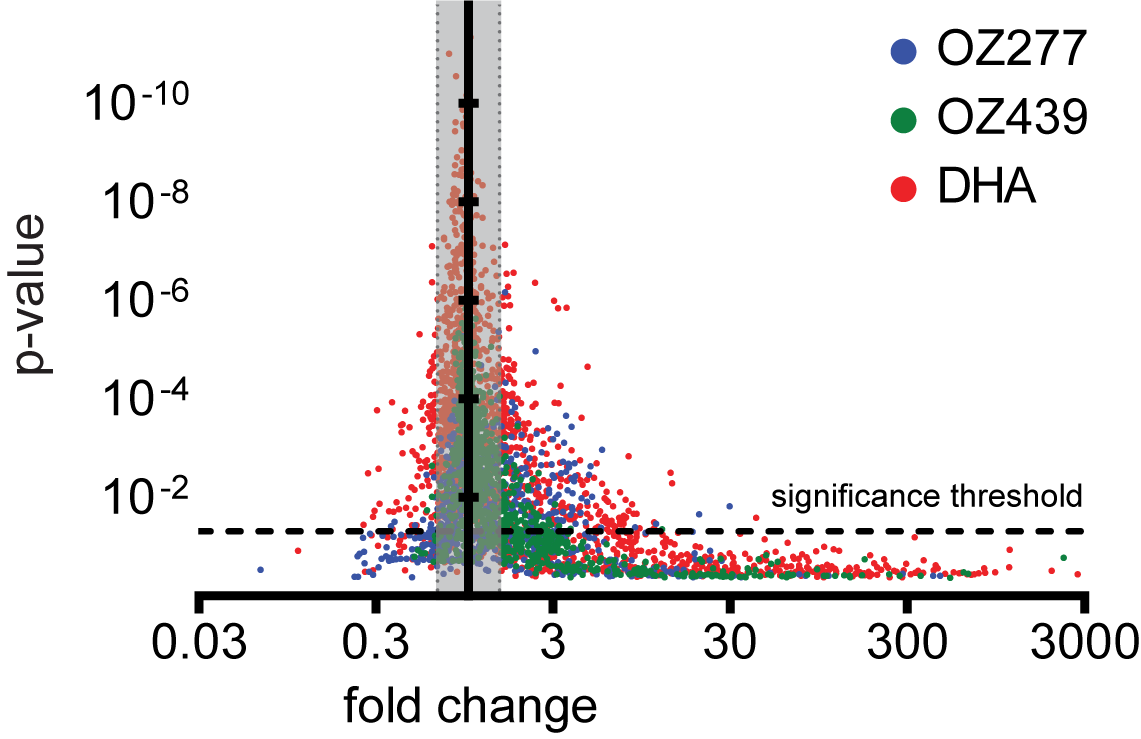

Supplement: S7 Fig — Proteins identified in at least three independent experiments following treatment of trophozoite stage parasites with OZ277 (1293 proteins, indicated by blue colour dots), OZ439 (1284 proteins, green dots) and DHA (1613 proteins, red dots). Trophozoite infected cultures were incubated with OZ277, OZ439 (both 300 nM) or DHA (100 nM) for 3 h. Proteins above the significance threshold (P-value ≤ 0.05) and outside the grey shaded area (fold change ≥ 1.5) were considered significant. (TIF) [file ppat.1008485.s007.tif]

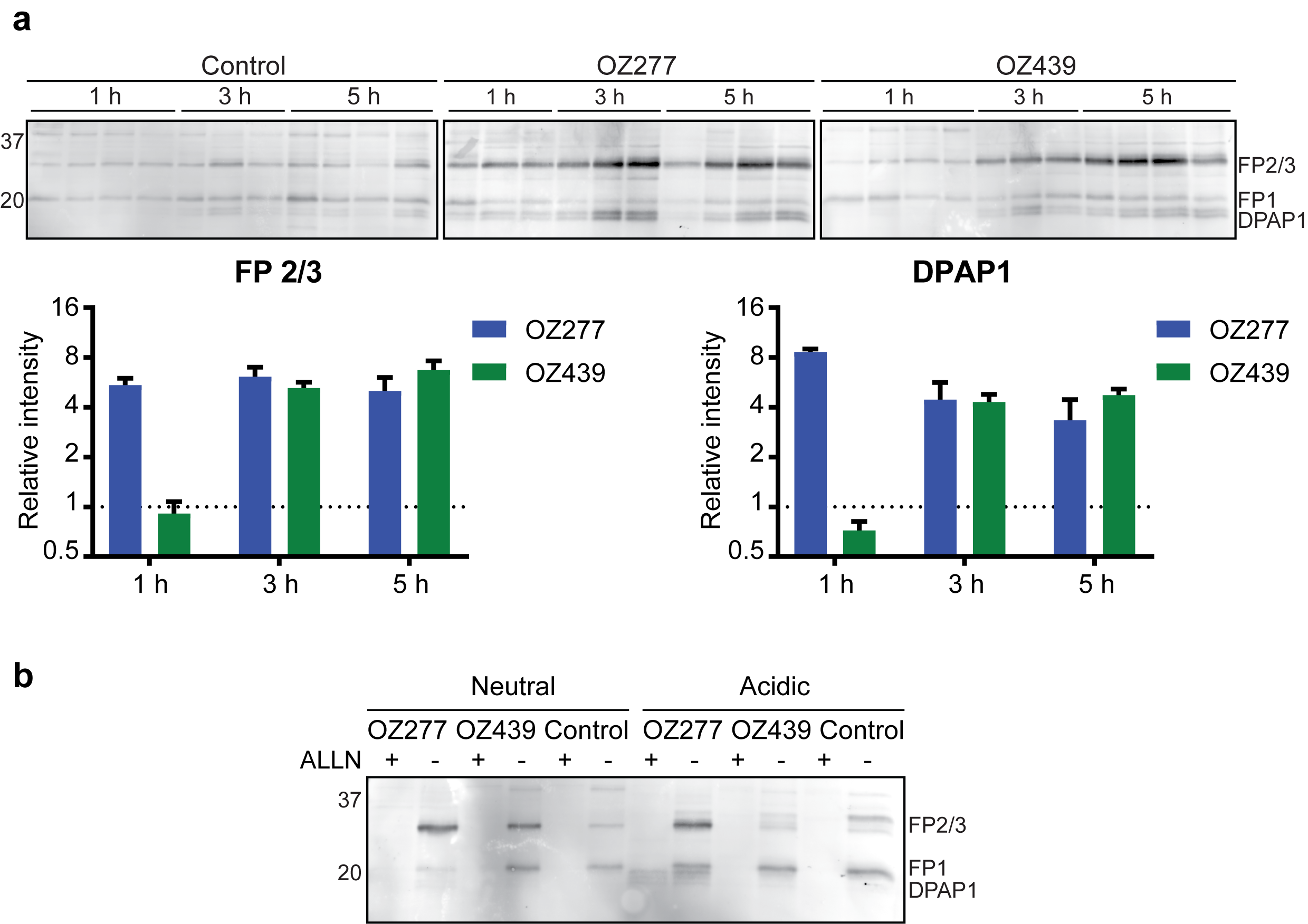

Supplement: S8 Fig — a, Cysteine protease activity and densitometric analysis of the DPAP1 and FP 2/3 signal after OZ277 and OZ439 treatment in P. falciparum trophozoite-stage parasites using DCG04 at pH 7.2 (neutral). Live parasites were treated for 1, 3 or 5 h with OZ277, OZ439 or DMSO (control) in 3–4 independent experiments and the saponin lysates were labelled with DCG04 to determine cysteine protease activity. DCG04 labelling was detected by blotting membranes with streptavidin-AF647 after SDS-PAGE and transfer. The lanes for each time point are independent drug treatments and represent at least three biological replicates per time point that were run on the same gel side-by-side. Densitometric analysis is shown relative to the untreated control at the corresponding time point (±SD). b, ALLN inhibition of cysteine protease activity under neutral (pH 7.2) and acidic (pH 5.5) conditions. Live parasites were treated for 3 h with OZ277, OZ439 or DMSO (control) and the saponin lysates were pre-incubated with (+) or without (-) the cysteine protease inhibitor ALLN (10 μM for 30 min) prior to labelling with DCG04. (TIF) [file ppat.1008485.s008.tif]

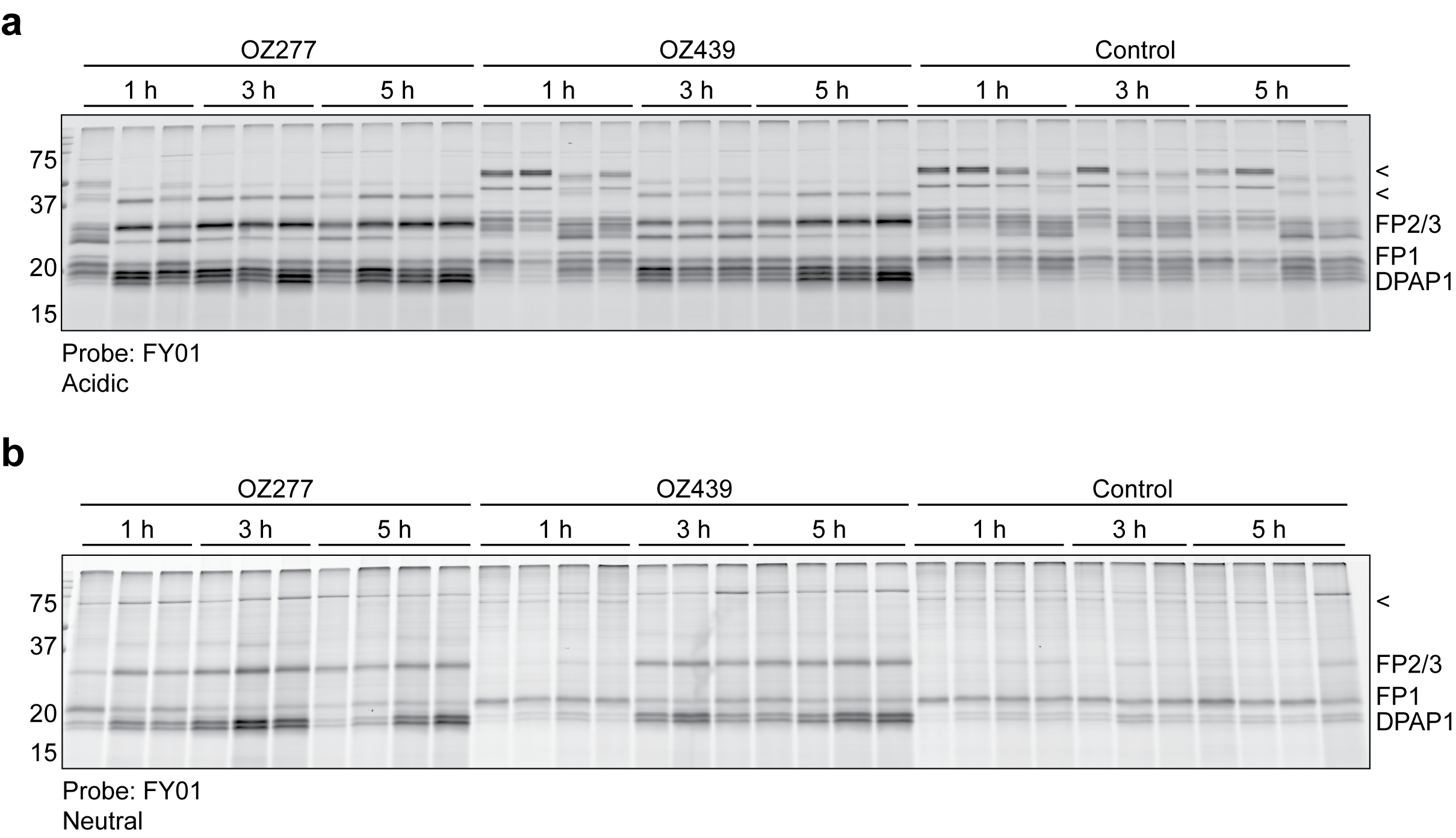

Supplement: S9 Fig — a-b, SDS-PAGE gel showing parasite cysteine protease activity after OZ277, OZ439 and DMSO (control) treatment in trophozoite-stage parasites using the FY01 probe, at pH 5.5 (acidic) (a) and pH 7.2 (neutral) (b). Live parasites were treated for 1, 3 or 5 h in 3–4 independent experiments. Treated parasites were saponin lysed and labelled with FY01 to determine cysteine protease activity. Proteins were resolved by SDS-PAGE and the gel was scanned for Cy5 fluorescence to detect probe labelling. For (a) and (b) the lanes for each time point are independent drug treatments and represent at least three biological replicates per time point that were run on the same gel side-by-side. < Unidentified bands. (TIF) [file ppat.1008485.s009.tif]

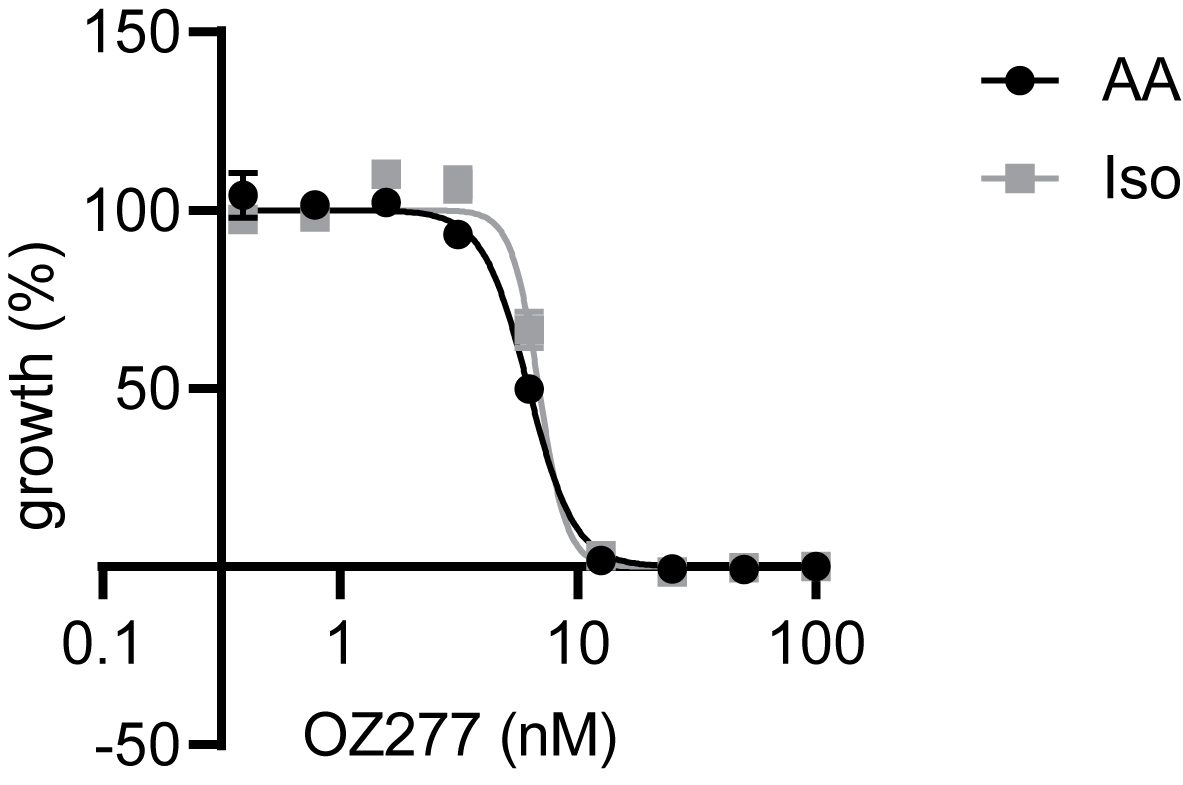

Supplement: S10 Fig — Trophozoite-stage parasites at 1% parasitaemia and 2% haematocrit cultured in either medium containing all amino acids (AA, black line) or isoleucine alone medium (Iso, grey line) were incubated with different concentration of OZ277 for 48 h and the 50% inhibitory concentration (IC50) determined using the SYBR green assay. The IC50 (nM) for OZ277 in parasites cultured in AA medium was 6.3 ± 1.6. The IC50 (nM) for OZ277 in parasites cultured in Iso medium was 6.6 ± 1.1. IC50 results are the mean and standard deviation of three biological repeats. A representative growth curve is shown. (TIF) [file ppat.1008485.s010.tif]

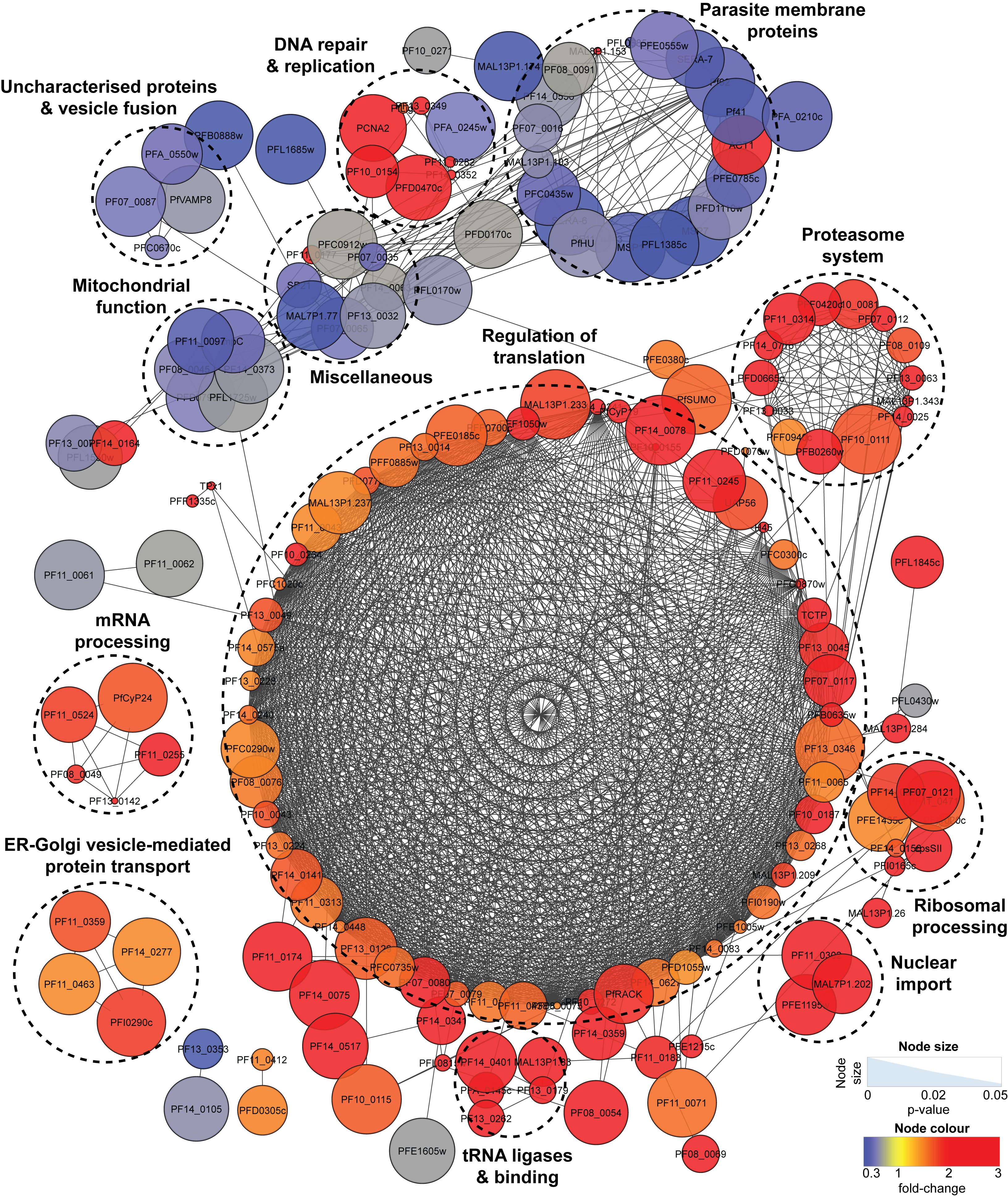

Supplement: S11 Fig — The network analysis was built using the STRINGdb interaction network analysis output (connectivity was based on experimental, database and co-expression evidence with a minimum interaction score of 0.7) in Cytoscape 3.6 with the ClusterONE algorithm. Node size represents P-value and node colour represents fold-change from at least three independent replicates. (TIF) [file ppat.1008485.s011.tif]

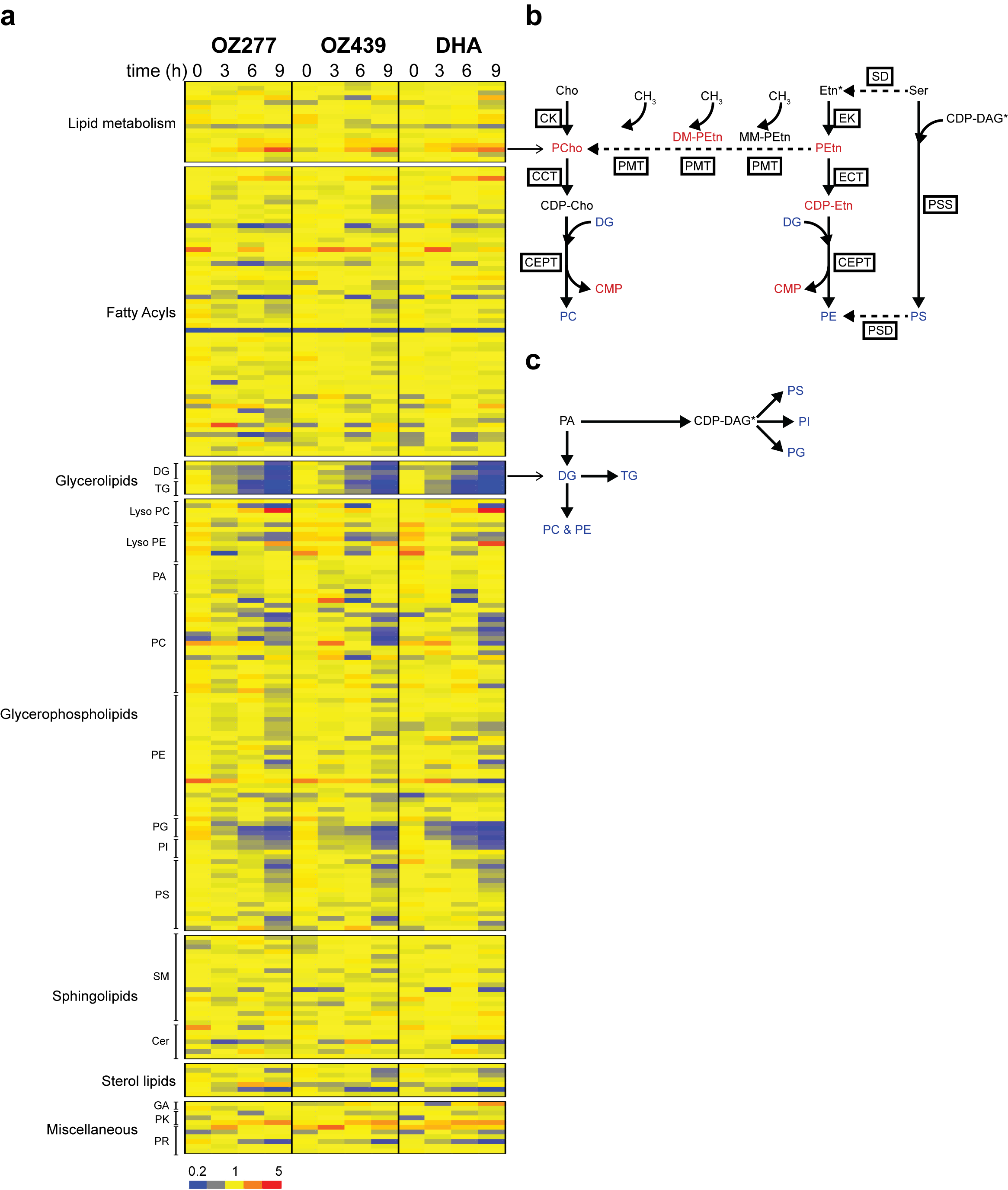

Supplement: S12 Fig — a, Heatmap showing the average fold change for all identified lipids and lipid metabolites at each time point after prolonged exposure to OZ277, OZ439 and DHA in trophozoite-stage P. falciparum parasites. Values represent the average of three biological replicates, expressed relative to the average untreated control value for that respective time point. b, De novo synthesis pathways for phosphatidylcholine (PC) and phosphatidylethanolamine (PE) in P. falciparum. Metabolites in red and blue were increased and decreased in abundance after drug treatment respectively. Enzyme names are shown in solid boxes. The dashed arrow represents an alternative route for the synthesis of PC from ethanolamine (Etn) in P. falciparum. c, Pathways for the synthesis of phospholipids and triglycerides from phosphatidic acid precursors. CCT, choline-phosphate cytidyltransferase; CDP-, cytidine-diphospho-; CEPT, choline/ethanolamine phosphotransferase; Cer, ceramide; Cho, choline; CK, choline kinase; CMP, cytidine monophosphate; DAG/DG, diglyceride; DM-, dimethyl-; ECT, ethanolamine-phosphate cyitidyltransferase; EK, ethanolamine kinase; Etn, ethanolamine; GA, gangliosides; MM-, monomethyl; PA, phosphatidic acid, PC, phosphatidylcholine; PCho, choline phosphate; PE, phosphatidylethanolamine; PEtn, ethanolamine phosphate; PG, phosphatidylglycerol; PI, phosphatidylinositol; PMT, phosphoethanolamine N-methyltransferase; PK, polyketides; PR, prenols; PS, phosphatidylserine; PSD, phosphatidylserine decarboxylase; PSS, phosphatidylserine synthase; SD, serine decarboxylase; SM, sphingomyelin; TG, triglyceride; TM-,trimethyl; *not detected. (TIF) [file ppat.1008485.s012.tif]

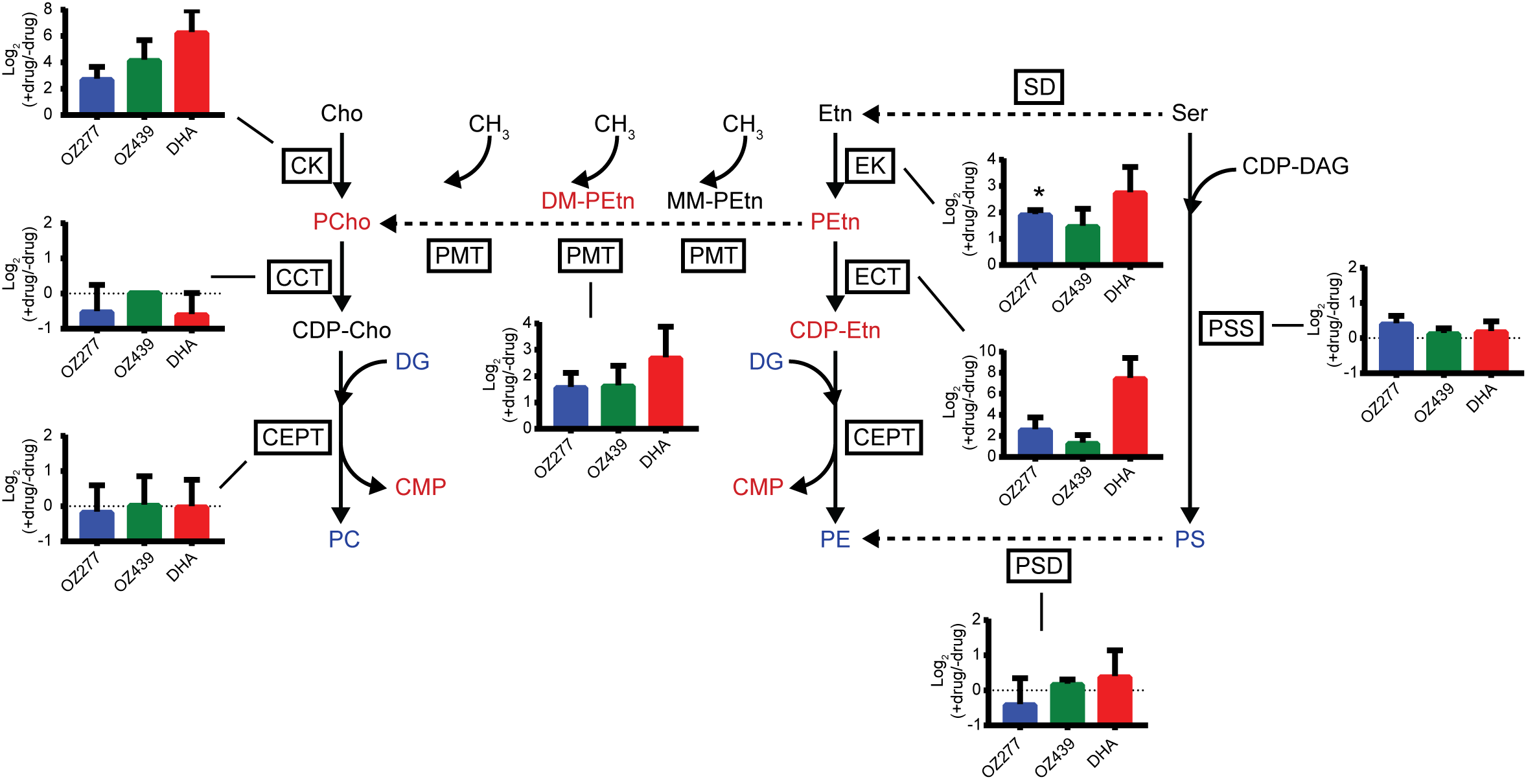

Supplement: S13 Fig — Values are the average fold change (± SD) relative to the untreated control of at least three biological replicates. Choline-phosphate cytidyltransferase (CCT) was only identified in one OZ439 experiment, therefore the mean from one experiment is shown. The last step in both the PC and PE branches of the Kennedy Pathways are catalysed by the enzyme choline/ethanolamine phosphotransferase (CEPT). For ease of representation, the abundance of CEPT is only shown for the PC branch of the pathway. * p-value < 0.05. CCT, choline-phosphate cytidyltransferase; CDP-, cytidine-diphospho-; CEPT, choline/ethanolamine phosphotransferase; Cer, ceramide; Cho, choline; CK, choline kinase; CMP, cytidine monophosphate; DAG/DG, diglyceride; DM-, dimethyl-; ECT, ethanolamine-phosphate cyitidyltransferase; EK, ethanolamine kinase; Etn, ethanolamine; GA, gangliosides; MM-, monomethyl; PA, phosphatidic acid, PC, phosphatidylcholine; PCho, choline phosphate; PE, phosphatidylethanolamine; PEtn, ethanolamine phosphate; PG, phosphatidylglycerol; PI, phosphatidylinositol; PMT, phosphoethanolamine N-methyltransferase; PK, polyketides; PR, prenols; PS, phosphatidylserine; PSD, phosphatidylserine decarboxylase; PSS, phosphatidylserine synthase; SD, serine decarboxylase; SM, sphingomyelin; TG, triglyceride; TM-,trimethyl. (TIF) [file ppat.1008485.s013.tif]

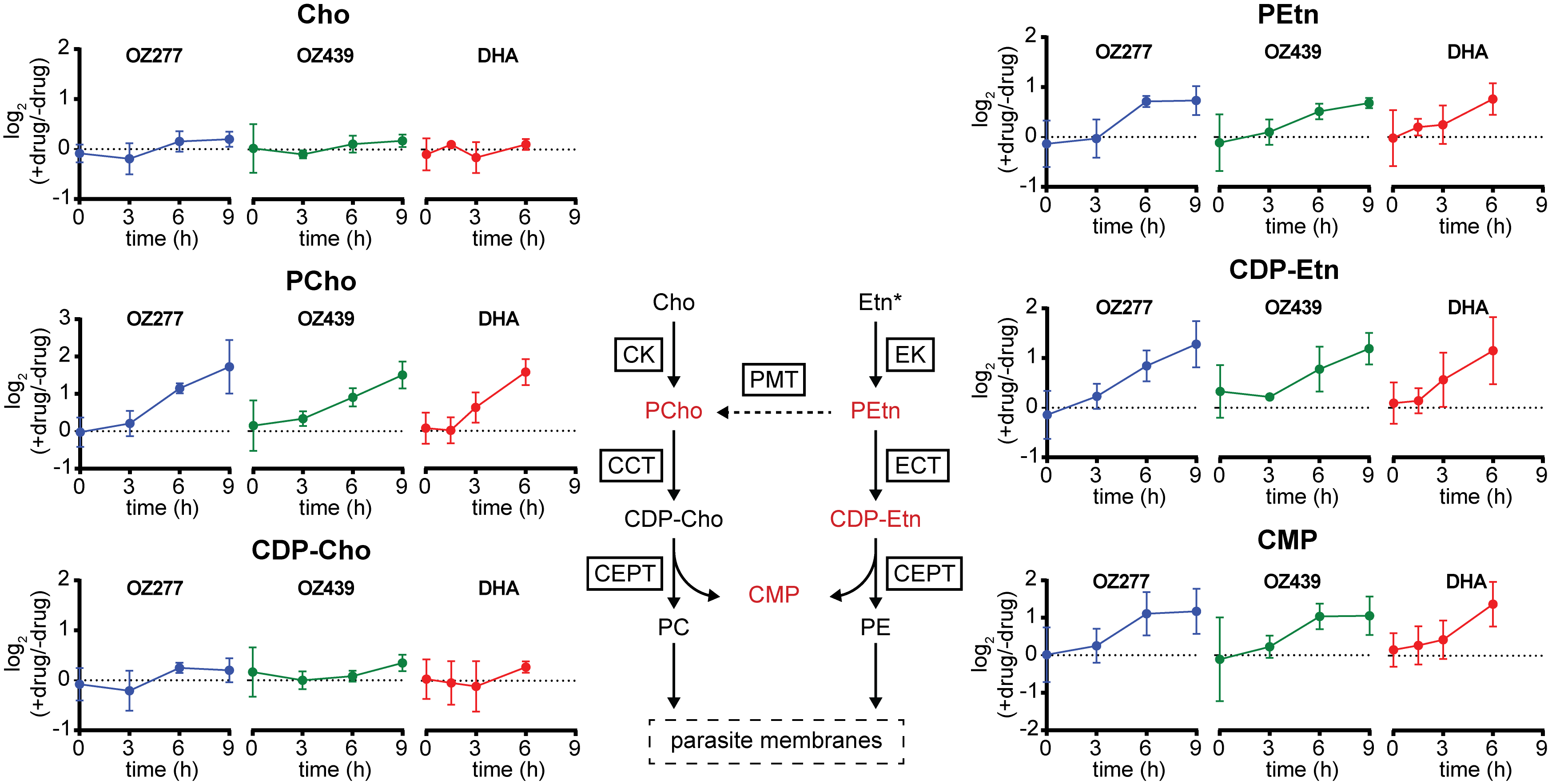

Supplement: S14 Fig — The abundances of each of the metabolites after treatment with OZ277 (blue), OZ439 (green) and DHA (red) are the average fold change (± SD) relative to the untreated control of four biological replicates. Metabolites in red were increased in abundance after drug treatment. Enzyme names are shown in solid boxes. The dashed arrow represents an alternative route for the synthesis of PC from ethanolamine (Etn) in P. falciparum parasites. CCT, choline-phosphate cytidyltransferase; CDP-, cytidine-diphospho-; CEPT, choline/ethanolamine phosphotransferase; Cho, choline; CK, choline kinase; CMP, cytidine monophosphate; ECT, ethanolamine-phosphate cytidyltransferase; EK, ethanolamine kinase; Etn, ethanolamine; PC, phosphatidylcholine; PE, phosphatidylethanolamine; PCho, choline phosphate; PEtn, ethanolamine phosphate; PMT, phosphoethanolamine N-methyltransferase; *not detected. (TIF) [file ppat.1008485.s014.tif]

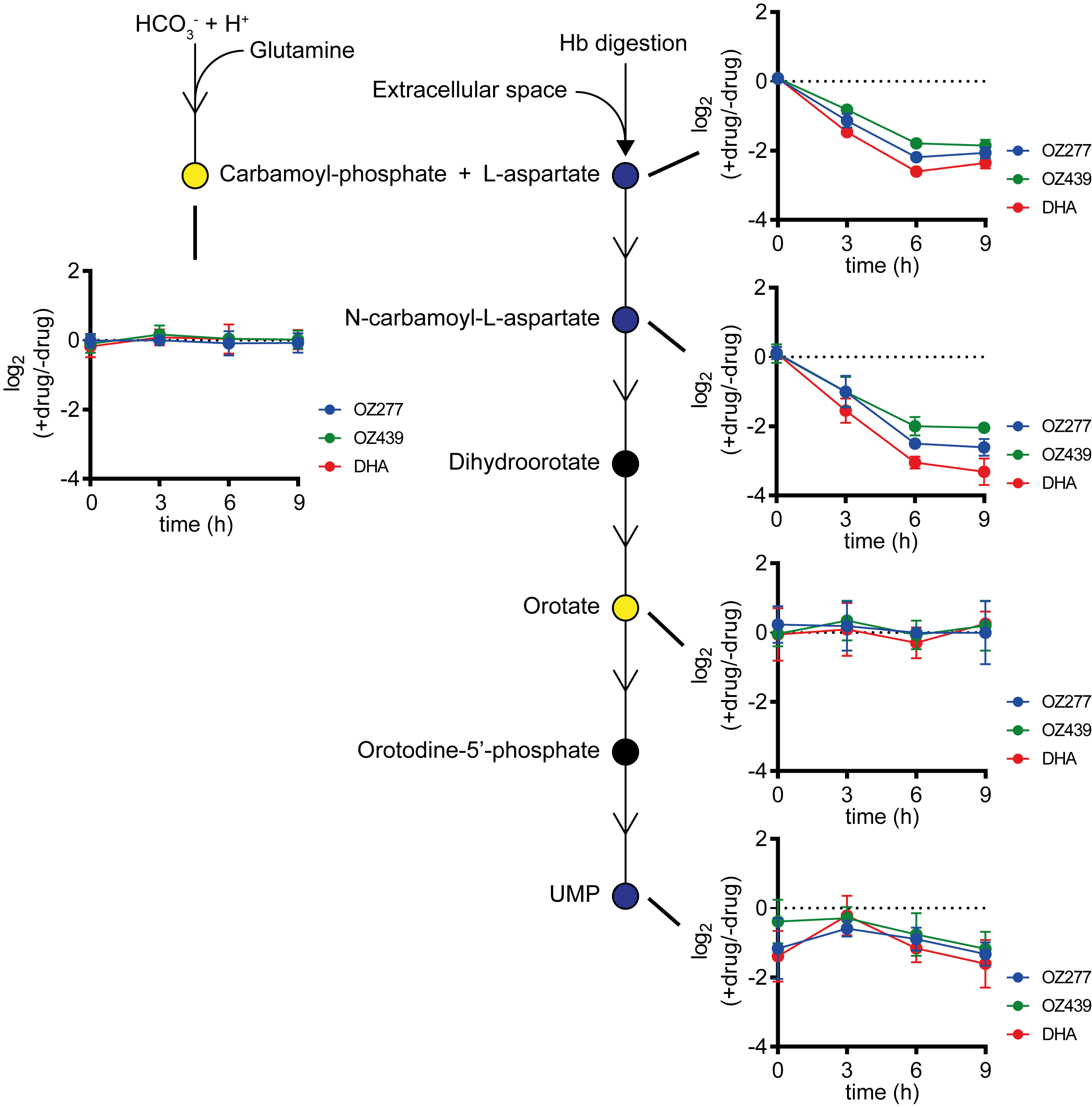

Supplement: S15 Fig — Values are the average fold change (± SD) relative to the untreated control of three biological replicates. For the pyrimidine biosynthesis pathway, blue circles represent metabolites that are decreased in abundance, yellow circles represent no change and black circles represent metabolites that were not detected. Hb, haemoglobin; UMP, uridine monophosphate. (TIF) [file ppat.1008485.s015.tif]

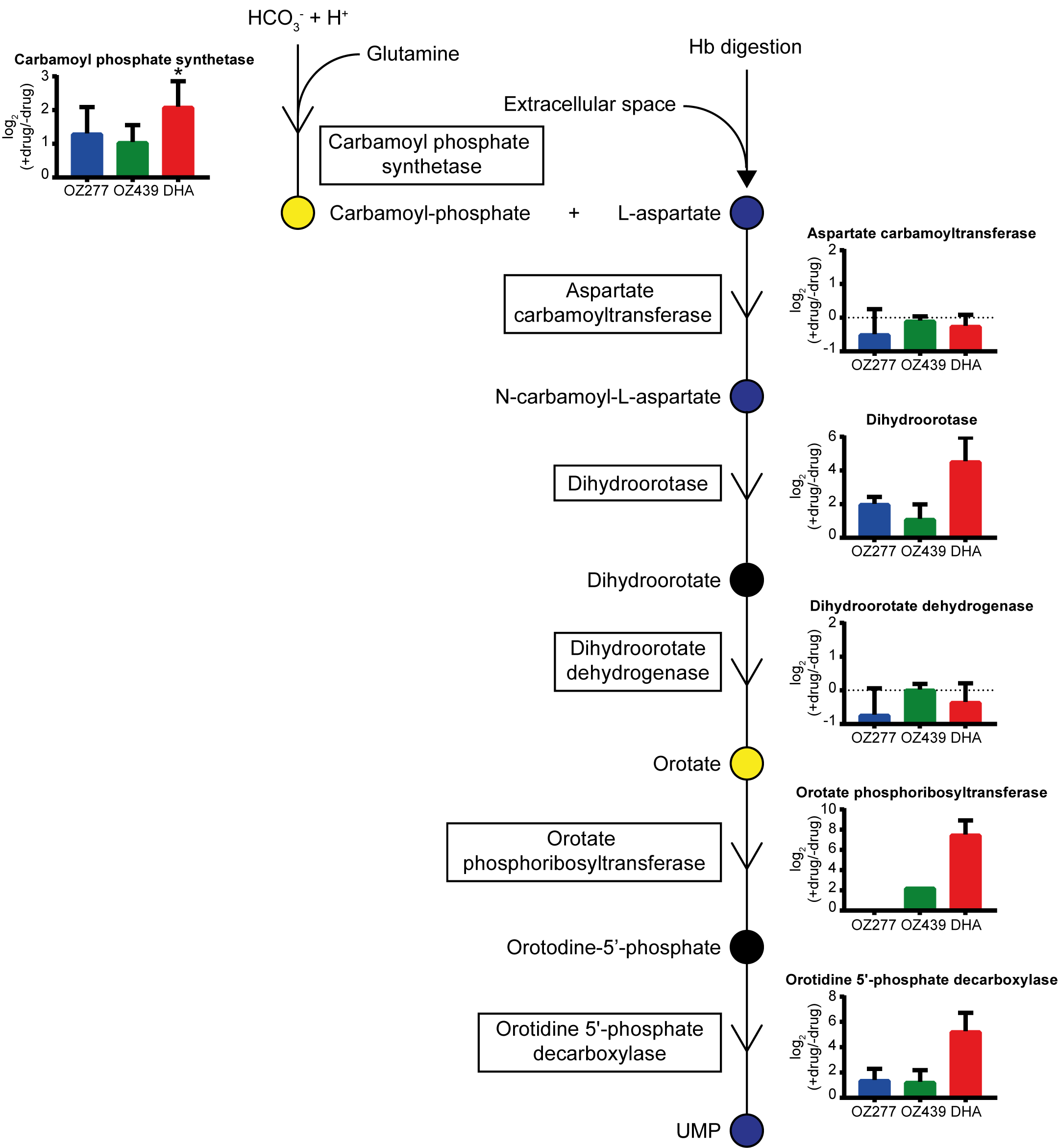

Supplement: S16 Fig — Values are the average fold change (± SD) relative to the untreated control of at least three biological replicates. Orotate phosphoribosyltransferase was not detected in OZ277 treated parasites and was only identified in one OZ439 experiment. * p-value < 0.05. (TIF) [file ppat.1008485.s016.tif]
